# Supplementary material for: FERN – a Java framework for stochastic simulation and evaluation of reaction networks
Source: BMC Bioinformatics. 2008 Aug 29;9:356. doi: 10.1186/1471-2105-9-356 (PMC2553347; doi:10.1186/1471-2105-9-356)
Supplement: Additional file 1 — FERN distribution, Version 1.3. This archive contains the FERN source code and binaries as well as documentation and example models in FernML and SBML. [file 1471-2105-9-356-S1.zip › fern/doc/javadoc/fern/simulation/observer/GnuPlotObserver.html]

GnuPlotObserver


---


|  |  |  |
| --- | --- | --- |
| **Overview** \| **Package** \| **Class** \| **Use** \| **Tree** \| **Deprecated** \| **Index** \| **Help** | |  |
| **PREV CLASS**   **NEXT CLASS** | **FRAMES**    **NO FRAMES**     **All Classes** |  |
| SUMMARY: NESTED \| FIELD \| CONSTR \| METHOD | DETAIL: FIELD \| CONSTR \| METHOD |  |


---


## fern.simulation.observer Interface GnuPlotObserver

**All Known Implementing Classes:**: AmountAtMomentObserver, AmountIntervalObserver, FireTypeObserver, IntervalObserver, LeapObserver, MultiAmountIntervalObserver, RandomNumberGeneratorCallObserver, ReactionIntervalObserver

---

``` public interface GnuPlotObserver ```

Interface for certain observer which can produce gnuplot data.

**Author:**
:   Florian Erhard

---

| **Method Summary** | |
| --- | --- |
| `String[]` | `getStyles()`             Gets the styles for the columns. |
| `GnuPlot` | `toGnuplot()`             Creates a new `GnuPlot` object and passes the actual observer data to it. |
| `GnuPlot` | `toGnuplot(GnuPlot gnuplot)`             Passes the actual observer data to a `GnuPlot` object. |

**Method Detail**

### getStyles

```
String[] getStyles()
```

:   Gets the styles for the columns. If you don't want styles, just return null!

    :   **Returns:**: styles for the columns

---


### toGnuplot

```
GnuPlot toGnuplot()
                  throws IOException
```

:   Creates a new `GnuPlot` object and passes the actual observer data to it.

    :   **Returns:**: the created `GnuPlot` object **Throws:**: `IOException` - if gnuplot could not be accessed

---


### toGnuplot

```
GnuPlot toGnuplot(GnuPlot gnuplot)
                  throws IOException
```

:   Passes the actual observer data to a `GnuPlot` object.

    :   **Parameters:**: `gnuplot` - the `GnuPlot` object to pass the data to **Returns:**: the `GnuPlot` object **Throws:**: `IOException` - if gnuplot could not be accessed


---


|  |  |  |
| --- | --- | --- |
| **Overview** \| **Package** \| **Class** \| **Use** \| **Tree** \| **Deprecated** \| **Index** \| **Help** | |  |
| **PREV CLASS**   **NEXT CLASS** | **FRAMES**    **NO FRAMES**     **All Classes** |  |
| SUMMARY: NESTED \| FIELD \| CONSTR \| METHOD | DETAIL: FIELD \| CONSTR \| METHOD |  |


---
